# Supplementary material for: An interactive murine single-cell atlas of the lung responses to radiation injury
Source: Nat Commun. 2023 Apr 28;14:2445. doi: 10.1038/s41467-023-38134-z (PMC10147670; doi:10.1038/s41467-023-38134-z)
Supplement: Supplementary file 1 — Supplementary_Figures [file 41467_2023_38134_MOESM1_ESM.pdf]

SUPPLEMENTARY FIGURES

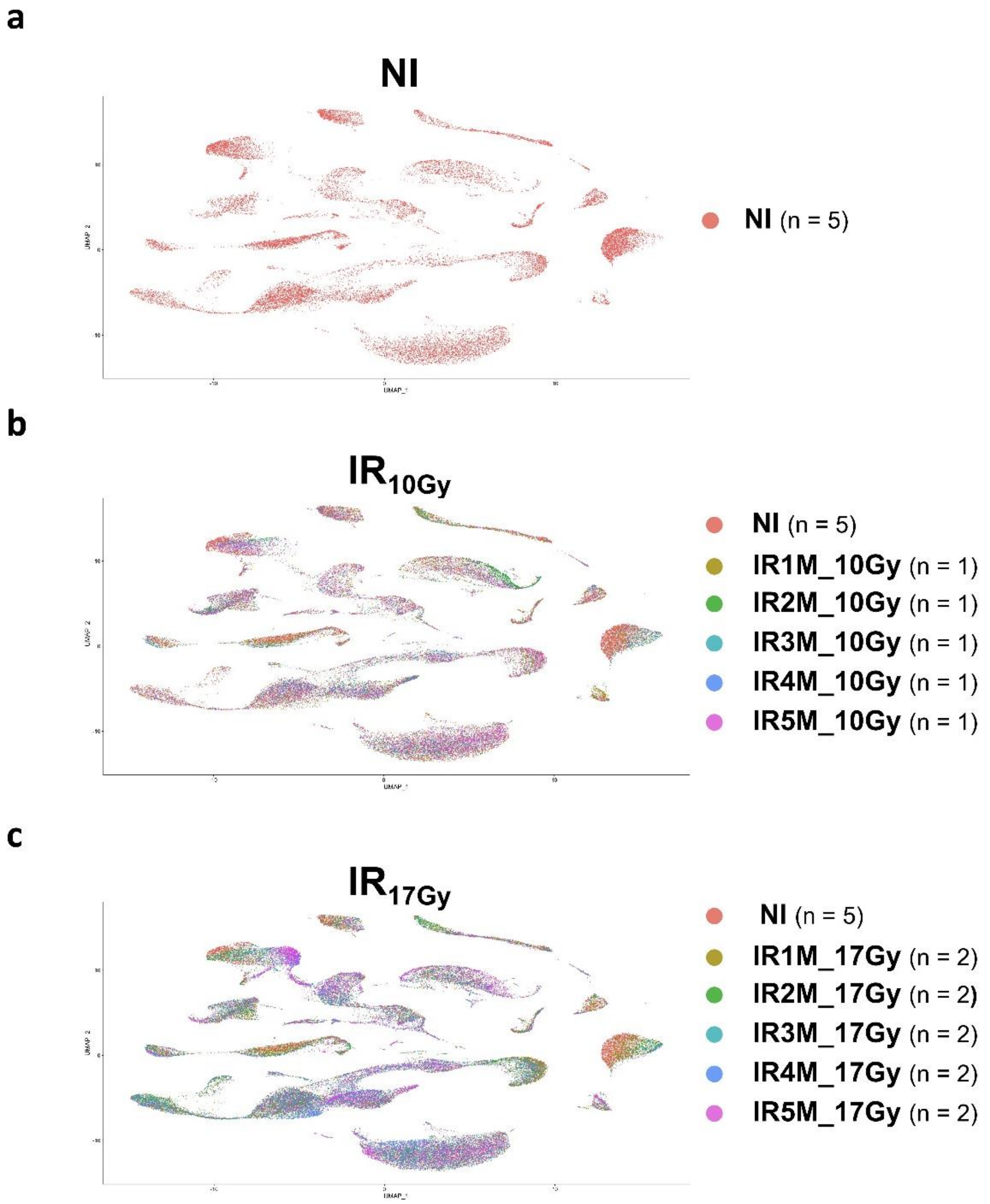

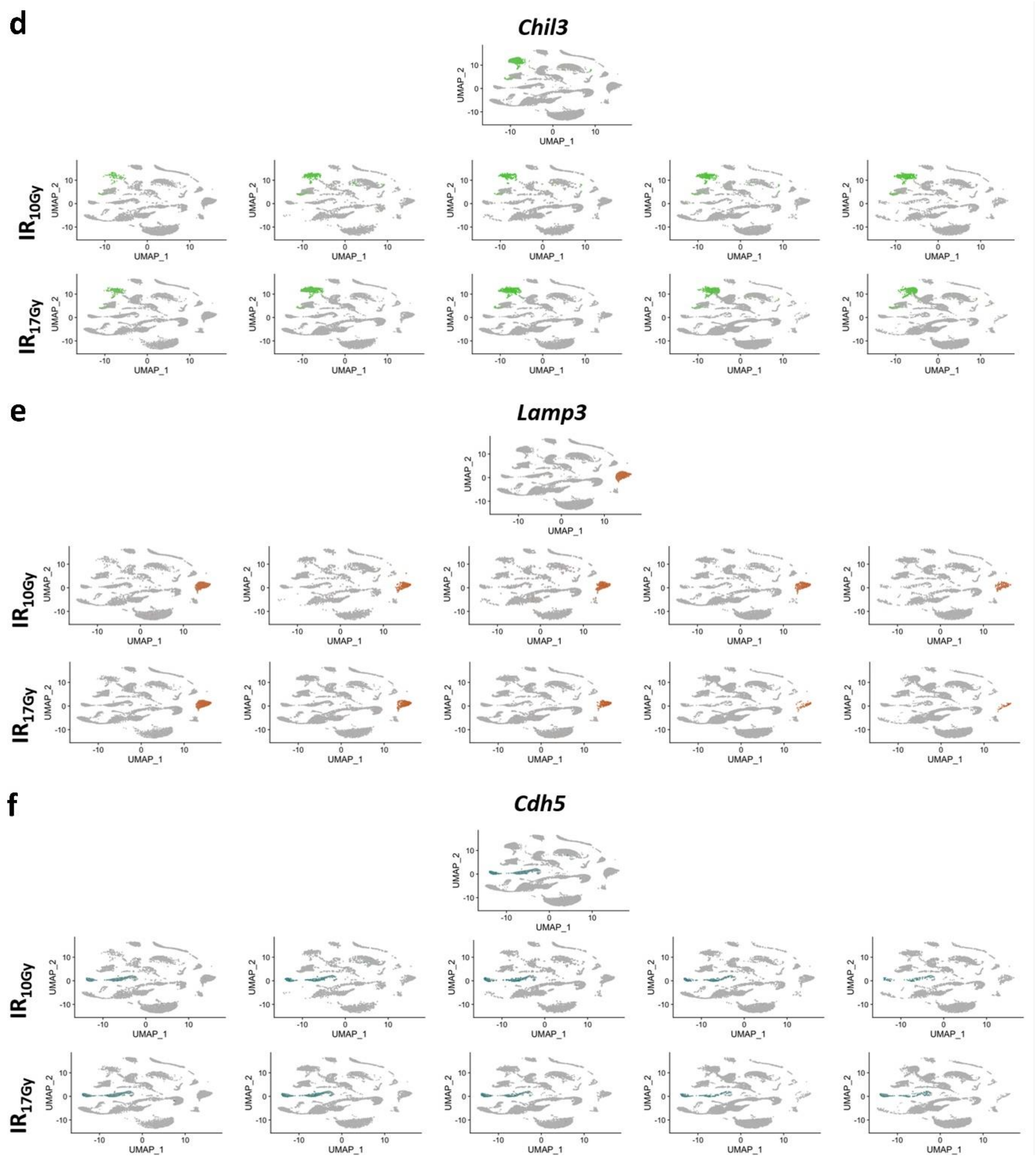

Supplementary Figure 1. Single-cell data visualization of the NI and IR lungs and cell type specific markers identification. UMAP visualization from **a** 5 NI samples alone (22,378 cells), **b** 5 NI samples and 5 IR_{10Gy} samples from 1 to 5M (one sample per time point) (26,360 cells) and **c** 5 NI samples and 10 IR_{17Gy} samples from 1 to 5M (two samples per time point) (54,131 cells). UMAP visualization of *Chil3* **d**, *Lamp3* **e** and *Cdh5* **f** expression in the NI samples and the different time points from 1 to 5 months after IR_{10Gy} (n =1) and IR_{17Gy} (n=2).

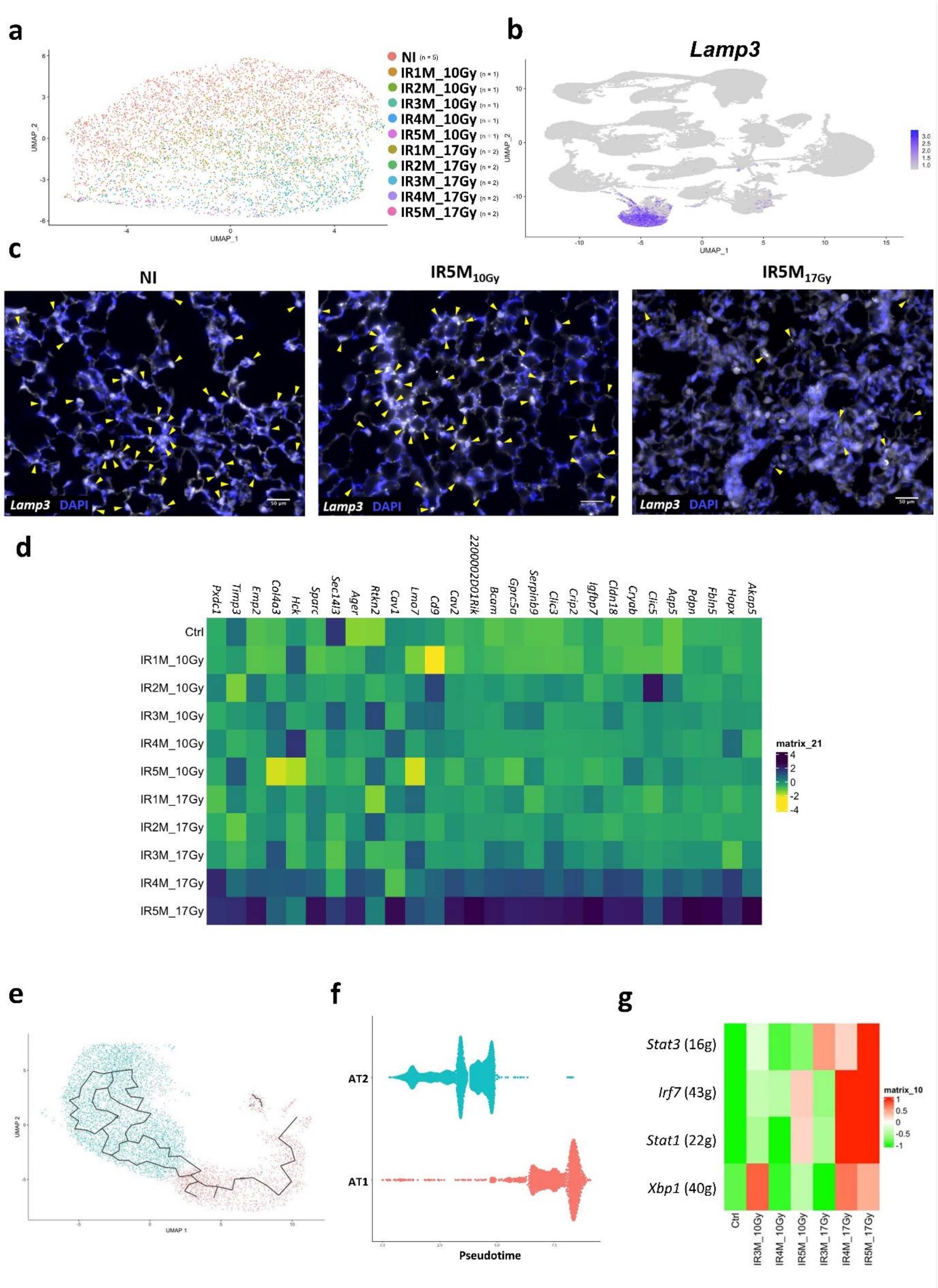
a
NI (n = 5)
IR1M_10Gy (n = 1)
IR2M_10Gy (n = 1)
IR3M_10Gy (n = 1)
IR4M_10Gy (n = 1)
IR5M_10Gy (n = 1)
IR1M_17Gy (n = 2)
IR2M_17Gy (n = 2)
IR3M_17Gy (n = 2)
IR4M_17Gy (n = 2)
IR5M_17Gy (n = 2)
UMAP_1
UMAP_2
b
Lamp3
c
NI
IR5M10Gy
IR5M17Gy
Lamp3 DAPI
d
Pxdc1 Timp3 Emp2 Col4a3 Hck Sparc Sec14l3 Ager Rtkn2 Cav1 Lmo7 Cd9 Cav2 2200002D01Rik Bcam Gprc5a Serpinb9 Clic3 Crip2 Igfbp7 Cldn18 Cryab Clic5 Aqp5 Pdpn Fbln5 Hopx Akap5
Ctrl
IR1M_10Gy
IR2M_10Gy
IR3M_10Gy
IR4M_10Gy
IR5M_10Gy
IR1M_17Gy
IR2M_17Gy
IR3M_17Gy
IR4M_17Gy
IR5M_17Gy
matrix_21
e
f
AT2
AT1
Pseudotime
g
Stat3 (16g)
Irf7 (43g)
Stat1 (22g)
Xbp1 (40g)
matrix_10
Ctrl
IR3M_10Gy
IR4M_10Gy
IR5M_10Gy
IR3M_17Gy
IR4M_17Gy
IR5M_17Gy

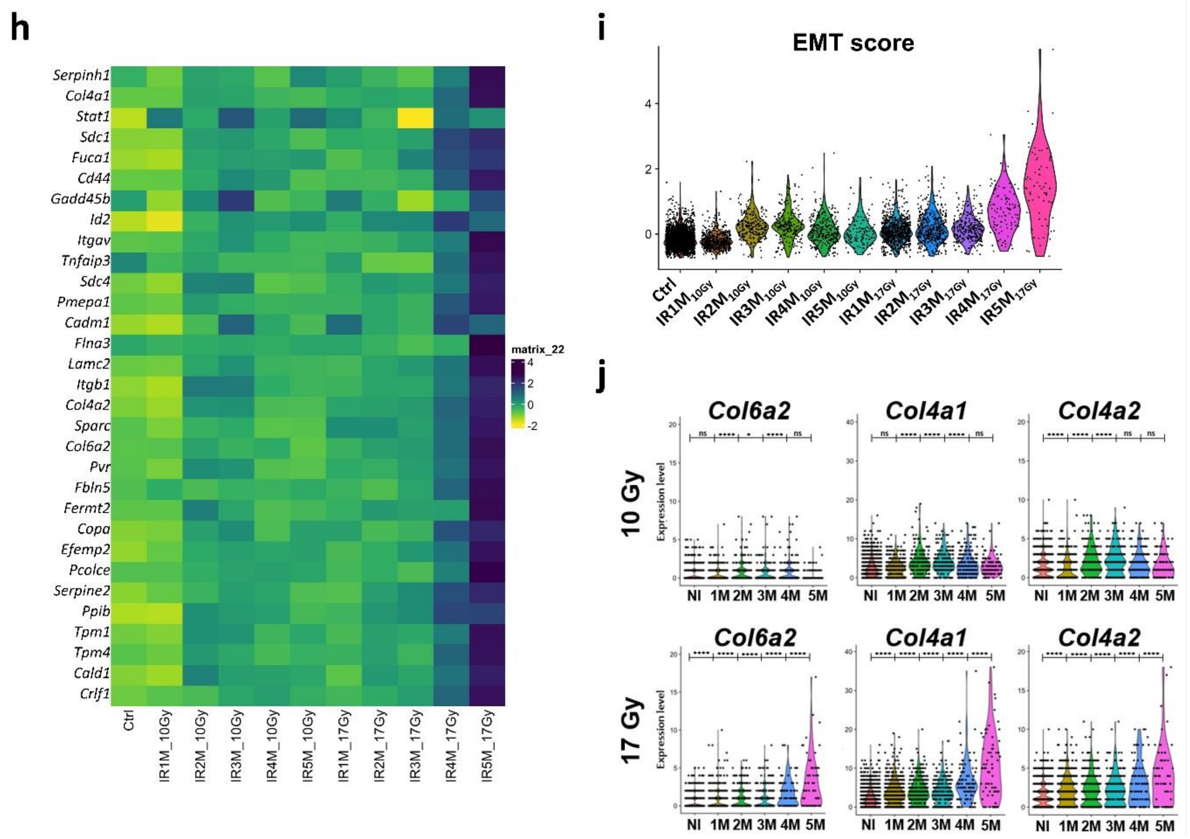

Supplementary Figure 2. Cellular and molecular characterization of AT2 cells. a UMAP visualization of the 5,550 NI (n = 5), IR$_{10Gy}$ (n = 1) and IR$_{17Gy}$ (n = 2) AT2 cells annotated by time point. **b** UMAP visualization of the expression of *Lamp3*. **c** *Lamp3* (white) staining in lung tissue sections from NI (n = 3), IR5M$_{10Gy}$ (n = 3) and IR5M$_{17Gy}$ (n = 5) mice. Sections were counterstained with DAPI (blue). Images are shown as a maximum intensity projection (16 z-stacks, 5 µm). Images were acquired using the tiles tool (5x5) on an apotome microscope with a 63X objective. Yellow arrows point at AT2 cells. Scale bars, 50 µm. **d** Heatmap of the expression of transdifferentiation related genes in the AT2 cells across the different samples. **e** Monocle trajectory analysis of the AT2 and AT1 clusters. **f** Monocle pseudotime analysis of the AT2 and AT1 cells using the marker genes of the transdifferentiating AT2 cluster 3 to order the cells. **g** Gene Regulatory network analysis of the AT2 cells in the NI samples and 3, 4 and 5 months after IR$_{10Gy}$ and IR$_{17Gy}$. **h** Heatmap of the expression of EMT genes in the AT2 cells across the different samples. **i** Violin plot showing the single cell score calculated based on the EMT expressed genes in the AT2 cells. **j** Violin plots of EMT genes expression in the AT2 cells in the NI samples and at the different time points after IR$_{10Gy}$ and IR$_{17Gy}$. (n/s, adjusted p-value > 0.05; *, adjusted p-value < 0.05; **, adjusted p-value < 0.01; ***, adjusted p-value < 0.001; ****, adjusted p-value < 0.0001).

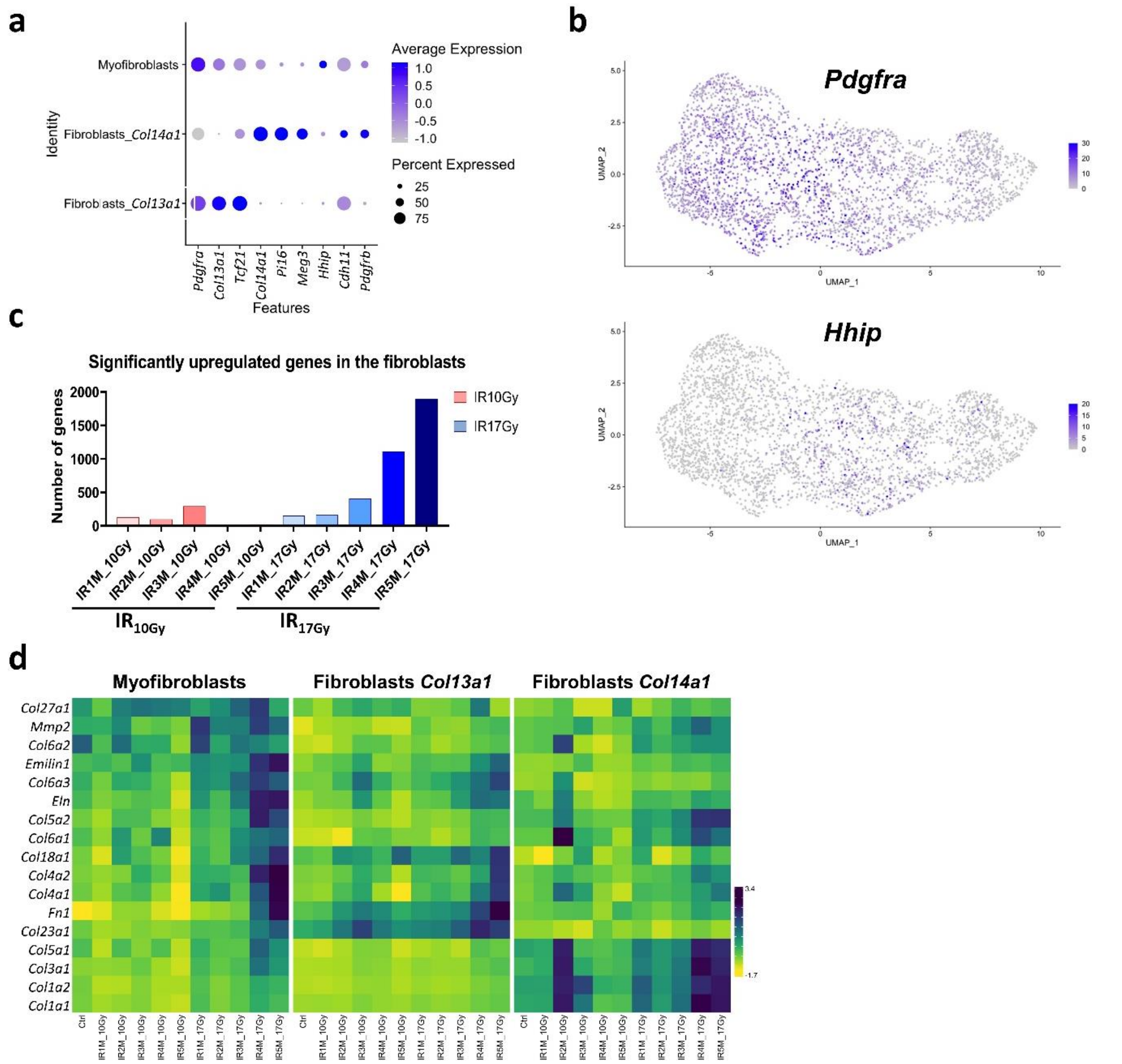

Supplementary Figure 3. Molecular characterization of the mesenchymal cells. a DotPlot of the expression of the marker genes used to identify the fibroblasts subpopulations: *Col13a1* and *Tcf21* for *Col13a1*+ fibroblasts; *Col14a1*, *Pi16* and *Meg3* for *Col14a1*+ fibroblasts; *Hhip*, *Cdh11* and *Pdgfrb* for myofibroblasts. **b** UMAP visualization of the expression of *Pdgfra* and *Hhip*. **c** Dynamics in the significantly upregulated genes in the fibroblast subpopulations compared to the NI samples at the different time points after IR10Gy and IR17Gy. **d** Heatmap of the expression of ECM related genes in the fibroblast subpopulations across the different samples.

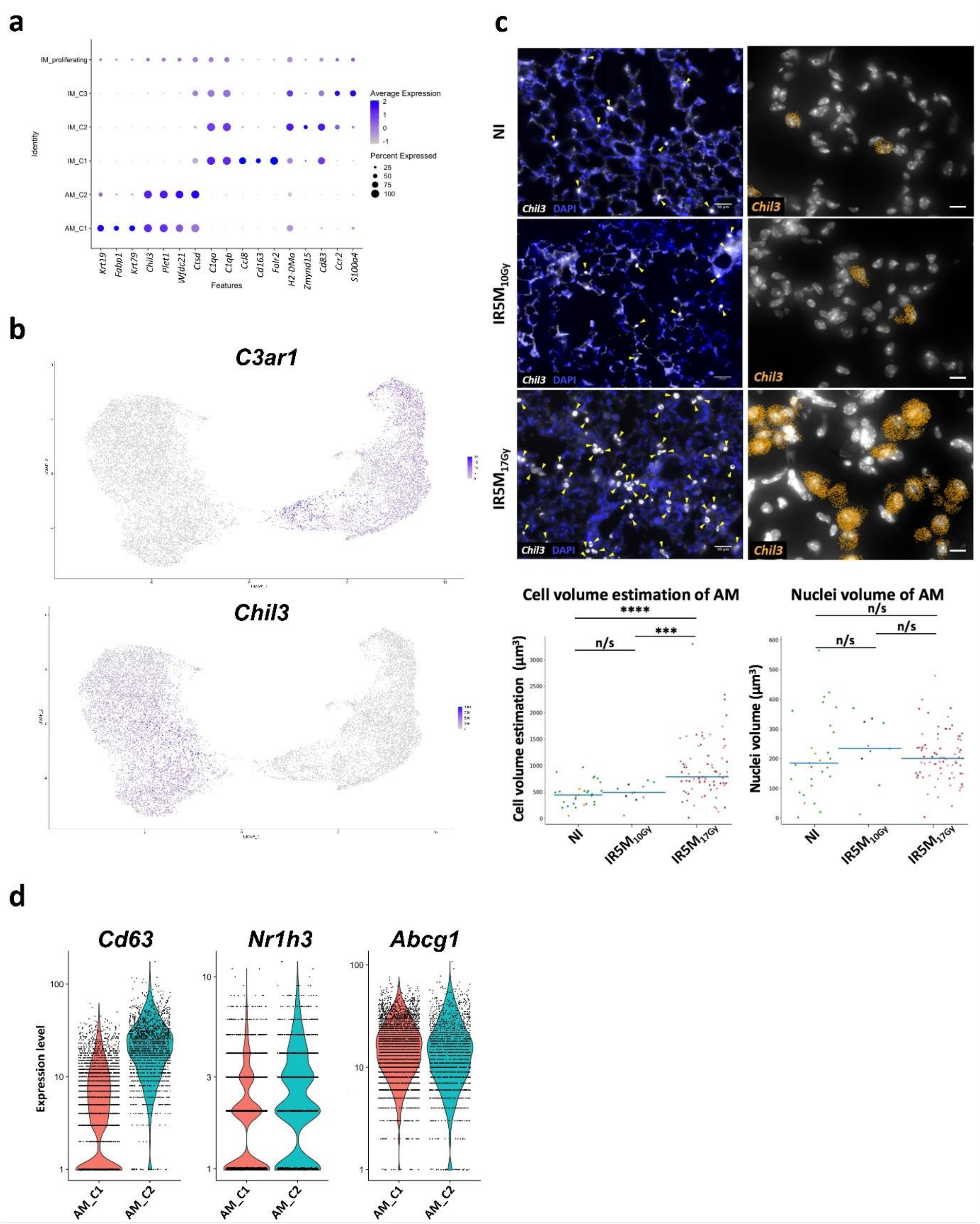

Supplementary Figure 4. Cellular and molecular characterization of the alveolar and interstitial macrophages. a DotPlot of the expression of the marker genes used to identify the different IM and AM subpopulations. **b** UMAP visualization of the expression of *C3ar1* and *Chil3*. **c** *Chil3* staining in lung tissue sections from NI, IR5M$_{10Gy}$ and IR5M$_{17Gy}$ mice. Left column: sections were counterstained with DAPI (blue). Images are shown as a maximum intensity projection (16 z-stacks, 5 µm). Images were acquired using the tiles tool (5x5) on an apotome microscope with a 63X objective. Yellow arrows point at AM. Scale bars, 50 µm. Right column: automatic *Chil3* mRNA detection with Big-FISH. Scale bars, 10 µm. Cell and nuclei volume estimation of the *Chil3*+ cells in NI, IR5M$_{10Gy}$ and IR5M$_{17Gy}$ lung

tissue sections. To compare two groups, the P-value was computed with the Mann–Whitney–Wilcoxon test (two-sided test) from scipy (n/s, adjusted p-value > 0.05; *, adjusted p-value < 0.05; **, adjusted p-value < 0.01; ***, adjusted p-value < 0.001; ****, adjusted p-value < 0.0001). Each dot represents one analyzed image. Each color per time point represents a different biological replicate (NI n = 3; $IR5M_{10Gy}$ n = 3; $IR5M_{17Gy}$ n = 5). **d** Violin plots of foam genes expression in the different AM subpopulations.

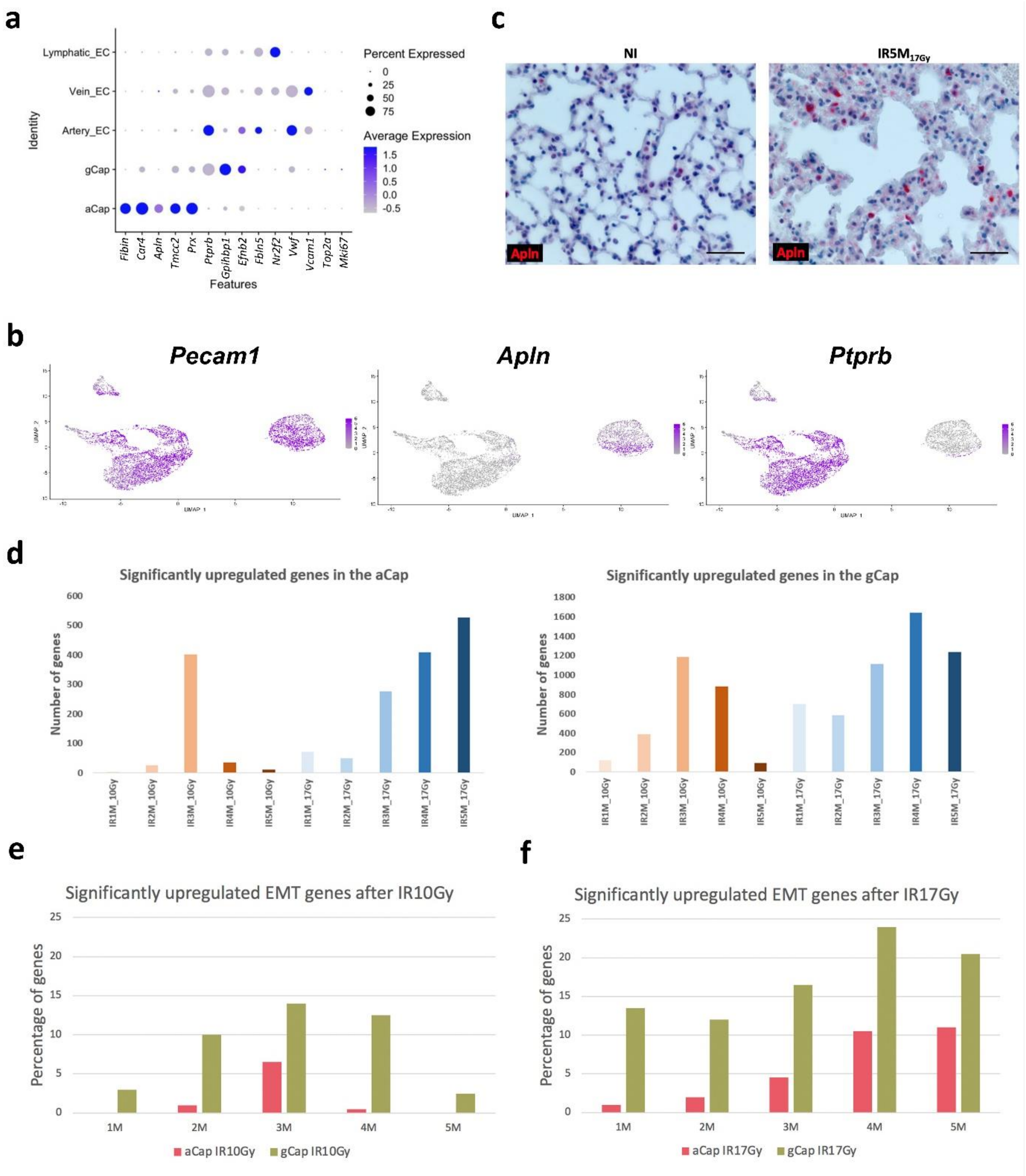

Supplementary Figure 5. Molecular profile of the different endothelial cell sub-compartments. a DotPlot of the expression of the marker genes used to identify the different EC subpopulations. **b** UMAP visualization of the expression of *Pecam1*, *Apln* and *Ptprb*. **c** Immunohistochemistry staining in NI (n = 3) and IR5M$_{17Gy}$ (n = 3) lung tissue sections using an anti-Apln antibody (red). Scale bars, 50 µm. **d** Dynamics in the significantly upregulated genes in the aCap and gCap compared to the NI samples at the different time points after IR$_{10Gy}$ and IR$_{17Gy}$. Percentage of the significantly upregulated EMT genes at the different time points after IR$_{10Gy}$ **e** and IR$_{17Gy}$ **f** in the aCap and gCap.

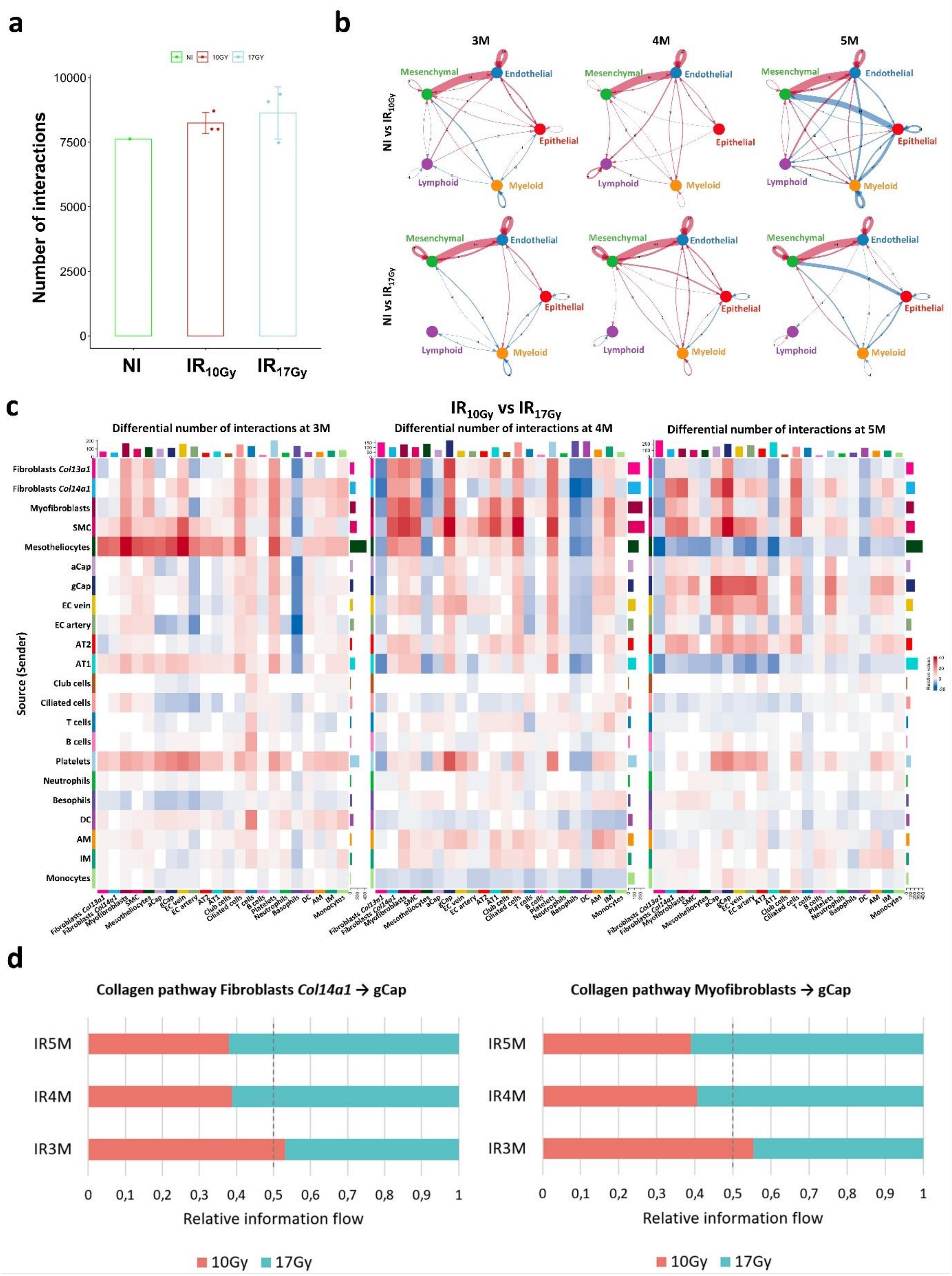
a
NI
10GY
17GY
Number of interactions
10000
7500
5000
2500
0
NI
IR10Gy
IR17Gy
b
3M
4M
5M
NI vs IR10Gy
NI vs IR17Gy
Mesenchymal
Endothelial
Epithelial
Lymphoid
Myeloid
c
IR10Gy vs IR17Gy
Differential number of interactions at 3M
Differential number of interactions at 4M
Differential number of interactions at 5M
Source (Sender)
Fibroblasts Col13a1
Fibroblasts Col14a1
Myofibroblasts
SMC
Mesotheliocytes
aCap
gCap
EC vein
EC artery
AT2
AT1
Club cells
Ciliated cells
T cells
B cells
Platelets
Neutrophils
Besophils
DC
AM
IM
Monocytes
d
Collagen pathway Fibroblasts Col14a1 → gCap
Collagen pathway Myofibroblasts → gCap
IR5M
IR4M
IR3M
0 0,1 0,2 0,3 0,4 0,5 0,6 0,7 0,8 0,9 1
Relative information flow
10Gy
17Gy

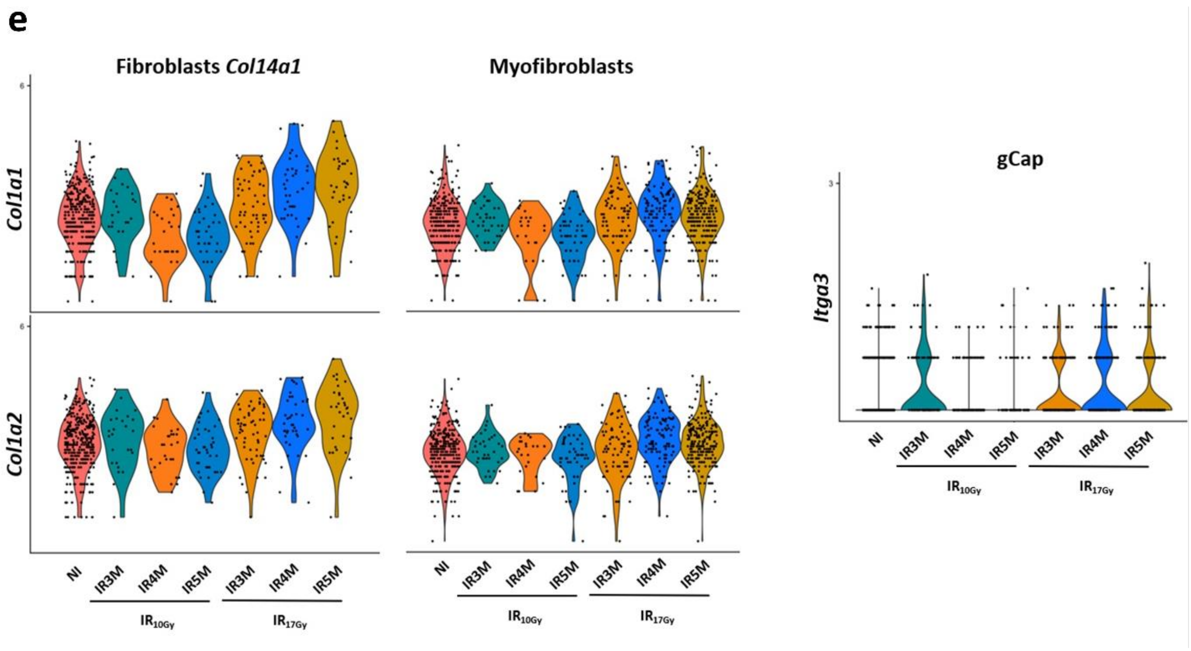

Supplementary Figure 6. Cell Chat interaction analysis of the major cell compartments. **a** Mean number of interactions in the NI samples (n=5 mice lungs were pulled together for the analysis) and the 3M, 4M and 5M IR_{10Gy} (n=3 mice lung) and IR_{17Gy} (n=6 mice lung; 2 samples per time point were pulled together for the analysis) samples. Error bars refer to the standard deviation of the data. **b** Circle plot showing the differential number of interactions between IR_{10Gy} and NI and IR_{17Gy} and NI in the main cellular compartments at 3M, 4M and 5M post-IR: mesenchymal, endothelial, epithelia, myeloid and lymphoid. Red (or blue) colored edges represent increased (or decreased) signaling in the IR compared to the NI. **c** Heatmap showing the differential number of interactions between IR_{10Gy} and IR_{17Gy} in all the different lung subpopulations at 3M, 4M and 5M post-IR. Red (or blue) represents increased (or decreased) signaling in the IR_{17Gy} compared to the IR_{10Gy}. The top-colored bar plot represents the sum of column of values displayed in the heatmap (incoming signaling). The right colored bar plot represents the sum of row of values (outgoing signaling). **d** Dynamics of the relative information flow of the Collagen pathway from 3 to 5 months post-IR from the Fibroblasts *Col14a1* and Myofibroblasts to the gCap. **e** Gene expression distribution of signaling genes related to the Collagen pathway in the NI samples and 3M, 4M and 5M after IR_{10Gy} and IR_{17Gy}: *Col1a1* and *Col1a2* ligands in Fibroblasts *Col14a1* and Myofibroblasts; *Itga3* receptor in gCap.

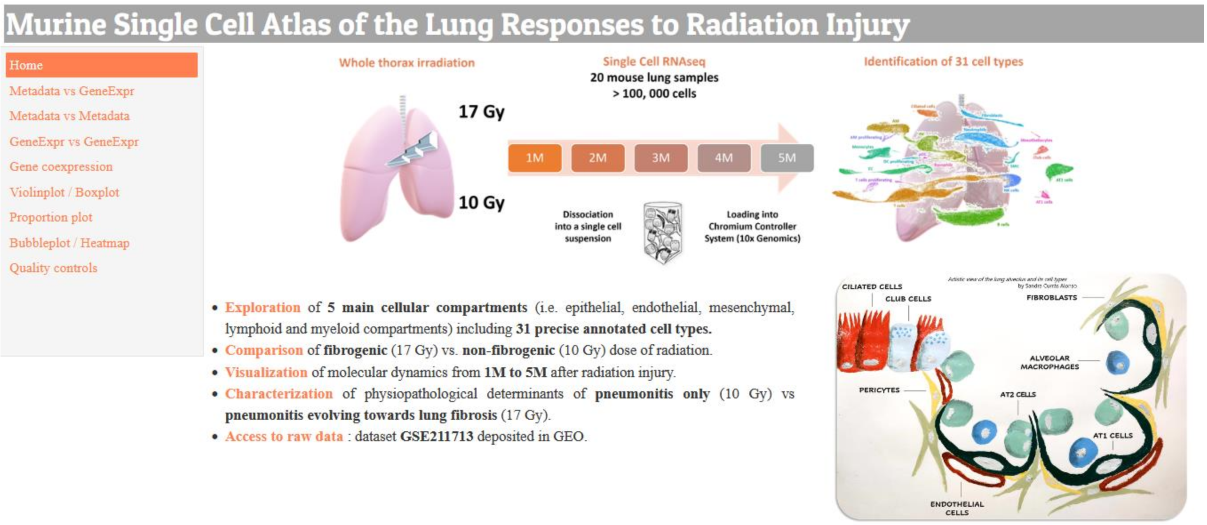

Supplementary Figure 7. **Interactive Mouse Radio-induced Pulmonary Fibrosis Atlas webpage.** Outline of the homepage of the interactive webpage containing the open-access transcriptomic data for all the scientific community. Bottom left image is an artistic view of the alveolus realized by Sandra Currás-Alonso. The website has been designed by Sophie Heinrich using the *ShinyCell* package.
