# Supplementary material for: An interactive murine single-cell atlas of the lung responses to radiation injury
Source: Nat Commun. 2023 Apr 28;14:2445. doi: 10.1038/s41467-023-38134-z (PMC10147670; doi:10.1038/s41467-023-38134-z)
Supplement: Supplementary file 3 — Reporting Summary [file 41467_2023_38134_MOESM3_ESM.pdf]

nature portfolio

Corresponding author(s): José-Arturo Londoño-Vallejo; Charles Fouillade

Last updated by author(s): Mar 17, 2023

Reporting Summary

Nature Portfolio wishes to improve the reproducibility of the work that we publish. This form provides structure for consistency and transparency in reporting. For further information on Nature Portfolio policies, see our Editorial Policies and the Editorial Policy Checklist.

Statistics

For all statistical analyses, confirm that the following items are present in the figure legend, table legend, main text, or Methods section.

n/a	Confirmed	
☐	☒	The exact sample size (*n*) for each experimental group/condition, given as a discrete number and unit of measurement
☐	☒	A statement on whether measurements were taken from distinct samples or whether the same sample was measured repeatedly
☐	☒	The statistical test(s) used AND whether they are one- or two-sided<br>*Only common tests should be described solely by name; describe more complex techniques in the Methods section.*
☒	☐	A description of all covariates tested
☒	☐	A description of any assumptions or corrections, such as tests of normality and adjustment for multiple comparisons
☒	☐	A full description of the statistical parameters including central tendency (e.g. means) or other basic estimates (e.g. regression coefficient) AND variation (e.g. standard deviation) or associated estimates of uncertainty (e.g. confidence intervals)
☒	☐	For null hypothesis testing, the test statistic (e.g. *F*, *t*, *r*) with confidence intervals, effect sizes, degrees of freedom and *P* value noted<br>*Give P values as exact values whenever suitable.*
☒	☐	For Bayesian analysis, information on the choice of priors and Markov chain Monte Carlo settings
☒	☐	For hierarchical and complex designs, identification of the appropriate level for tests and full reporting of outcomes
☒	☐	Estimates of effect sizes (e.g. Cohen's *d*, Pearson's *r*), indicating how they were calculated

Our web collection on statistics for biologists contains articles on many of the points above.

Software and code

Policy information about availability of computer code

Data collection	Count matrices were generated using Cell Ranger (3.0.2). smFISH images were acquired using ZEN software (Blue edition) from Carl Zeiss
Data analysis	Single cell RNAseq analysis were performed in R (4.0.5) with Seurat (4.0.1). To remove RNA contaminant, we applied SOupX (1.5.2). Trajectory analysis were done using Monocle 2 and Monocle 3. Gene regulary network analysis used SCENIC package (1.3.1). Cell-cell interactions were analysed with CellChat (1.1.3)

For manuscripts utilizing custom algorithms or software that are central to the research but not yet described in published literature, software must be made available to editors and reviewers. We strongly encourage code deposition in a community repository (e.g. GitHub). See the Nature Portfolio guidelines for submitting code & software for further information.

Data

Policy information about availability of data

All manuscripts must include a data availability statement. This statement should provide the following information, where applicable:

- Accession codes, unique identifiers, or web links for publicly available datasets
- A description of any restrictions on data availability
- For clinical datasets or third party data, please ensure that the statement adheres to our policy

The scRNA-seq datasets generated during the current study have been deposited in the Gene Expression Omnibus (GEO) repository, with the accession code GSE211713 - (https://www.ncbi.nlm.nih.gov/geo/query/acc.cgi?acc=GSE211713). Processed data can be explored through an interactive web interface (https://lustra.shinyapps.io/shinyapp/).

Human research participants

Policy information about studies involving human research participants and Sex and Gender in Research.

Reporting on sex and gender	*Use the terms sex (biological attribute) and gender (shaped by social and cultural circumstances) carefully in order to avoid confusing both terms. Indicate if findings apply to only one sex or gender; describe whether sex and gender were considered in study design whether sex and/or gender was determined based on self-reporting or assigned and methods used. Provide in the source data disaggregated sex and gender data where this information has been collected, and consent has been obtained for sharing of individual-level data; provide overall numbers in this Reporting Summary. Please state if this information has not been collected. Report sex- and gender-based analyses where performed, justify reasons for lack of sex- and gender-based analysis.*
Population characteristics	*Describe the covariate-relevant population characteristics of the human research participants (e.g. age, genotypic information, past and current diagnosis and treatment categories). If you filled out the behavioural & social sciences study design questions and have nothing to add here, write "See above."*
Recruitment	*Describe how participants were recruited. Outline any potential self-selection bias or other biases that may be present and how these are likely to impact results.*
Ethics oversight	*Identify the organization(s) that approved the study protocol.*

Note that full information on the approval of the study protocol must also be provided in the manuscript.

Field-specific reporting

Please select the one below that is the best fit for your research. If you are not sure, read the appropriate sections before making your selection.

☒ Life sciences ☐ Behavioural & social sciences ☐ Ecological, evolutionary & environmental sciences

For a reference copy of the document with all sections, see nature.com/documents/nr-reporting-summary-flat.pdf

Life sciences study design

All studies must disclose on these points even when the disclosure is negative.

Sample size	No sample-size calculation was performed.
Data exclusions	For single cell RNAseq analysis, low quality cells were removed based on stringent criteria such as number of transcripts, proportion of mitochondrial genes.
Replication	Whenever it was possible, replication was performed with different mice analyzed and results were reproduced.
Randomization	Mice were allocated to each experimental groups randomly.
Blinding	For technical reasons, blinding during data acquisition was not possible but for data analysis of smFISH images, investigator in charge of the analysis was blind to the different experimental groups.

Reporting for specific materials, systems and methods

We require information from authors about some types of materials, experimental systems and methods used in many studies. Here, indicate whether each material, system or method listed is relevant to your study. If you are not sure if a list item applies to your research, read the appropriate section before selecting a response.

Materials & experimental systems

n/a	Involved in the study	
☐	☒	Antibodies
☒	☐	Eukaryotic cell lines
☒	☐	Palaeontology and archaeology
☐	☒	Animals and other organisms
☒	☐	Clinical data
☒	☐	Dual use research of concern

Methods

n/a	Involved in the study	
☒	☐	ChIP-seq
☒	☐	Flow cytometry
☒	☐	MRI-based neuroimaging

Antibodies

Antibodies used	Apelin (Apln) polyclonal antibody (Invitrogen, #PA5-114860, Lot WD3261456A)
Validation	https://www.thermofisher.com/antibody/product/Apelin-Antibody-Polyclonal/PA5-114860

Animals and other research organisms

Policy information about studies involving animals; ARRIVE guidelines recommended for reporting animal research, and Sex and Gender in Research

Laboratory animals	The study used wild type C57BL6J mice
Wild animals	The study did not involve wild animals
Reporting on sex	The study used classical model of thoracic radiation injury using female mice. thus, no sex-based analysis was performed.
Field-collected samples	The study did not involve samples collected from the field.
Ethics oversight	Experimental procedures were specifically approved by the ethics committee of the Institut Curie CEEA-IC #118 (Authorization number APAFIS#5479-201605271 0291841 given by National Authority) in compliance with the international guidelines.

Note that full information on the approval of the study protocol must also be provided in the manuscript.
